# Weak Prediction Power of the Framingham Risk Score for Coronary Artery Disease in Nonagenarians

**DOI:** 10.1371/journal.pone.0113044

**Published:** 2014-11-13

**Authors:** Josef Yayan

**Affiliations:** Department of Internal Medicine, Division of Pulmonary, Allergy, and Sleep Medicine, Saarland University Medical Center, Homburg/Saar, Germany; University of Texas MD Anderson Cancer Center, United States of America

## Abstract

**Background:**

Coronary artery disease (CAD) is caused by an acute myocardial infarction and is still feared as a life-threatening heart disease worldwide. In order to identify patients at high risk for CAD, previous studies have proposed various risk assessment scores for the prevention of CAD. The most commonly used risk assessment score for CAD worldwide is the Framingham Risk Score (FRS). The FRS is used for middle-aged people; hence, its appropriateness has not been demonstrated to predict the likelihood of CAD occurrence in very elderly people. This article examines the possible predictive value of FRS for CAD in very elderly people over 90 years of age.

**Methods:**

Data on all patients over 90 years of age who received a cardiac catheter were collected from hospital charts from the Department of Internal Medicine, Saarland University Medical Center, and HELIOS Hospital Wuppertal, Witten/Herdecke University Medical Center, Germany, within a study period from 2004 to 2013. The FRSs and cardiovascular risk profiles of patients over 90 years of age with and without CAD after cardiac catheterization were compared.

**Results:**

One hundred and seventy-five (91.15%, mean age 91.51±1.80 years, 74 females [42.29%]; 95% confidence interval [CI], 0.87–0.95) of a total 192 of the very elderly patients were found to have CAD. Based on the results of our study, the FRS seems to provide weak predictive ability for CAD in very elderly people (P = 0.3792).

**Conclusion:**

We found weak prediction power of FRS for CAD in nonagenarians.

## Introduction

Coronary artery disease (CAD) is the most common heart disease and hides the high risk for the cause for the development of acute myocardial infarction [1]. Numerous studies and international and national clinical practice guidelines have proven that CAD is caused by the manifestation of atherosclerosis in coronary arteries [2]–[10]. According to data from epidemiological studies, CAD has an increasingly high mortality rate around the world [11]. For this reason, the prediction of CAD risk has gained significant attention in the medical science community worldwide. The identification of risk factors for CAD is a basic requirement for establishing possible targeted medical therapy for the primary and secondary prevention of CAD. Therefore, several national and international guidelines and recommendations for preventing CAD were previously published after identifying the risk factors for CAD [12]–[15]. There are still ongoing efforts and attempts to improve the risk assessment methods for the prediction of CAD. To achieve this goal, several risk prediction scores for CAD have been developed in recent years [16]. Five or 10 risk assessments for CAD have been assumed worldwide according to the recommendations of the guidelines [17]–[18]. Currently available CAD risk prediction scores are mostly based on multivariable regression analysis deduced from the Framingham Heart Study [19] in which the traditional risk factors for CAD are taken into consideration such as age, cholesterol levels, blood pressure, smoking, and body weight [20]–[21]. The Framingham Risk Score (FRS) provides an estimation of the probability of an individual developing CAD in 10 years to detect high-risk persons and to take preventive actions [22]. Based on data obtained through the FRS calculations, high-risk patients should be treated, according to the guidelines' recommendations, with lipid-lowering medication and aspirin in the primary prevention of CAD [23], [24]. FRS and other presently common risk estimation scores are designed for people in middle age [20], [25], [26]. The mean age in the FRS was 49 years old and people younger than 30 years and older than 74 years of age were not considered [20], [27]. Present risk prediction with the FRS might operate less effectively in elderly compared to middle-aged persons, and various traditional risk factors have a weak association with CAD risk in the elderly; for example, hypercholesterolemia is a strong cardiovascular risk factor in middle-aged individuals, but not in the elderly [27], [28]. Thus, new questions arise as to whether the FRS could be used to estimate cardiovascular risk for very elderly people over 90 years of age. We conducted the present investigation to better understand the FRS as an eligible prediction system for CAD in very elderly people over 90 years of age. Therefore, we collected data on all patients of this age group with CAD according to the International Classification of Disease from the hospital database at the Department of Internal Medicine, Saarland University Medical Center, and HELIOS Hospital Wuppertal, Witten/Herdecke University Medical Center, Germany. We used a risk assessment tool based on information from the Framingham Heart Study to calculate the FRS after confirming the presence or absence of CAD by performing cardiac catheterization to examine the FRS as an eligible scoring system for very elderly people. The variety of calculated FRS for CAD in people over 90 years of age were age, gender, systolic blood pressure, total cholesterol, high density lipoproteins (HDL), tobacco smoking, and former smoking. CAD diagnosis was made only after cardiac catheterization. The FRS for CAD was compared in patients older than 90 years of age after excluding CAD by performing cardiac catheterization. Only once we have identified the cardiovascular risk factors of CAD can we develop appropriately tailored therapies for all patients to take precautions against CAD.

## Materials and Methods

### Ethics Statement

All patients' data were anonymized prior to analysis. Due to the retrospective nature of the study protocol, the Medical Association of Saarland's Institutional Review Board approved this study and waived the need for informed consent.

### Patients

In this study, the FRS for CAD was retrospectively examined in patients over 90 years of age using hospital chart data at the Department of Internal Medicine, Saarland University Medical Center, and HELIOS Hospital Wuppertal, Witten/Herdecke University Medical Center, during the study period from 2004 to 2013.

The FRS for CAD in the last decade of a patient's life was considered theoretically, assuming that the average life expectancy would be 100 years of age. For the control group, the last highest decade of life was chosen to avoid any distortion in the data analysis due to age. The last highest decade of life refers to patients over 90 years old. Thus, the study population was composed of very elderly patients over 90 years of age diagnosed with CAD, and the control group was composed of elderly patients over 90 years of age without CAD, as determined after cardiac catheterization. All patients older than 90 years who were treated at the internal medicine emergency rooms or in one of the internal departments of the two hospitals were included after receiving a cardiac catheter in this study. Patients over 90 years who were treated in other departments or had no cardiac catheter were excluded from this study.

The FRS assessment tool from the National Heart, Lung, and Blood Institute (NHLBI) in Bethesda, Maryland [12], [29] was used to calculate FRSs in very elderly people over 90 years of age after CAD was confirmed to be present or absent by cardiac catheterization. The study population received a point score based on the categorical values of age, total cholesterol, high-density lipoprotein cholesterol (HDL), blood pressure, diabetes, and smoking [30]. Former smokers were considered smokers when calculating the FRS in this study. All patients' 0–5 Risk Scores for CAD were determined, giving one point for existing cardiovascular risk factors. A score of 0 means no risk for CAD, 1 very low risk, 2 moderate risk, 3 increased risk, 4 high risk, and 5 very high risk for CAD. Once CAD was detected or excluded by cardiac catheterization, we calculated FRSs retrospectively using the data from very elderly people over 90 years of age collected from the two hospital charts data, assuming that the examined cardiovascular risk factors in this study existing at the time of detection or exclusion of CAD would also have existed 10 years ago.

CAD has been defined as a chronic disease of the coronary arteries characterized by the manifestation of atherosclerosis with variable coronary artery stenosis, resulting in myocardial ischemia. CAD symptoms are classified as stable angina and ACS. ACS is a collective term for unstable angina, NSTEMI, and STEMI. The main symptom of coronary insufficiency is angina pectoris; it involves localized retrosternal pain triggered by physical and mental stress. Such chest pain may spread to the neck, lower jaw, shoulder, back, left arm to the fingertips, or the upper abdomen. Unstable angina refers to angina pectoris occurring for the first time, as well as a worsening of pain intensity and the duration of episodes.

NSTEMI was described for unstable angina and myocardial infarction with an increase in cardiac enzymes such as high-sensitivity cardiac troponin T without ST-segment elevation on an electrocardiogram. A 12-lead electrocardiogram was used at rest for the temporary recording of the sum of the heart's electrical activity to diagnose STEMI or cardiac arrhythmias in all patients. Typical ST-segmental change in the electrocardiogram for STEMI was considered ST-segment elevation>0.1 mV in at least one derivation. The diagnosis of CAD was made after cardiac catheterization. The quantitative determination of high-sensitivity troponin T in human plasma was measured after sample collection in lithium heparin SARSTEDT Monovette 4.7 ml (orange top) using a standard immunoturbidimetric assay on the COBAS INTEGRA system (the normal value is less than 14 pg/ml) after conservation from cardiac troponin T on September 2, 2010. A second measurement of high-sensitivity troponin T was carried out three hours after the first blood sample.

Prior to this, from January 1, 2004, to September 2, 2010, electrochemiluminescence immunoassay (ECLIA) was performed to determine cardiac troponin T (the normal value is less than 0.01 µg/l) on Elecsys 2010 and cobas e 411 immunoassay analyzers (Roche Diagnostics Ltd., Mannheim, Germany).

The classification of CAD was performed in each case according to the latest edition of the International Classification of Disease (ICD I25.11–I25.13) from 2004 to 2013. Coronary artery disease injuries were categorized as 1-, 2, or 3-vessel. Further, examiners visually estimated the degree of stenosis diameter as a percentage of the cardiac catheter, as per the stenosis morphology classification recommendations of the American College of Cardiology/American Heart Association [31].

We compared the cardiovascular risk factors in accordance with the guidelines of the International Atherosclerosis Society [32], such as arterial hypertension, diabetes mellitus, hypercholesterolemia, hyperlipidemia, obesity, tobacco smoking, and former smoking in very elderly patients over 90 years of age with and without CAD (ICD I25.0–I25.10) after completing cardiac catheterization.

Arterial hypertension was described as a condition in which the blood pressure of the arterial vascular system is chronically elevated. According to the World Health Organization (WHO) [33], hypertension can be diagnosed with a systolic blood pressure of at least 140 mmHg or a diastolic blood pressure of at least 90 mmHg (ICD I10.90). The manifestation of hypertension was described as a known history of hypertension where the patient has been treated with drugs. Blood pressure was measured by the indirect method following Riva-Rocci, 24-hour blood pressure measurement and blood pressure monitors DINAMAP (GE Medical Systems Information Technologies Ltd., Freiburg, Germany).

Diabetes mellitus (ICD E14.90) was diagnosed as a chronic metabolic disease based on an absolute or relative lack of insulin with elevated blood glucose levels when fasting values were more than 126 mg/dl or when, occasionally, a measured value above 200 mg/dl was detected in the serum of the patients. Blood glucose was determined in the serum of all patients using the SARSTEDT serum Monovette 4.7 ml (brown top) blood collection system with a multifly blood collection needle.

As hypercholesterolemia (ICD E78.0) is considered a lipid metabolic disorder characterized by elevated blood cholesterol levels higher than 200 mg/dl, total cholesterol and HDL (reference range 35–65 mg/dl) were measured after 12 hours of fasting in all patients in the plasma after blood collection in lithium heparin SARSTEDT Monovette 4.7 ml (orange top) with a multifly blood collection needle as an enzymatic colorimetric test using Roche cobas c 701 systems (Roche Diagnostics Ltd., Mannheim, Germany). Hyperlipidemia (ICD E78.2–E78.3) is mainly diagnosed through elevated triglycerides in the blood plasma of patients. The reference range for hypertriglyceridemia has been specified as>200 mg/dl in blood plasma after 12 hours of fasting following blood collection in lithium heparin SARSTEDT Monovette 4.7 ml (orange top) with a multifly blood collection needle as an enzymatic colorimetric test using Roche cobas c 701 systems (Roche Diagnostics Ltd., Mannheim, Germany).

Obesity (ICD E66.99) was defined as the excessive growth of adipose tissue in the body. The transition from overweight to obese was achieved with a body mass index (BMI) of 30. The designation of obese was made by calculating the body weight in kilograms divided by height in meters squared. Nicotine abuse (ICD F17.1) was designated as the abusive consumption of products that contain nicotine, including cigarettes, cigars, and other tobacco products. The study population was categorized into smokers, former smokers, and non-smokers. The quantification of tobacco smoking by measuring packs per year was not considered in this study because the harmful effect of nicotine was not the focus of the research.

We analyzed acute and chronic comorbidities as predisposing factors for the development of CAD in elderly people. In addition, the length of the study and control groups' hospital stays was compared.

### Statistical analysis

The data were expressed in proportion, mean, and standard deviation wherever appropriate. We calculated 95% confidence intervals (CIs) for the total number of patients with CAD. Odds ratios were calculated for the presence of cardiovascular risk factors for CAD, sex, and acute and chronic comorbidities. Gender difference was calculated using the chi-square test for two independent standard normal variables of two probabilities. A calculation of the chi-square test for four independent standard normal variables of two probabilities was used to compare the association between cardiovascular risk factors and stable angina, unstable angina, NSTEMI, or STEMI. Fisher's exact test for three variables of two probabilities was calculated for cardiovascular risk factors in different forms of CAD. One-way analysis of variance (ANOVA) for independent samples was performed to compare the duration of hospital stays, BMI, body height, body weight, cholesterol, HDL, and systolic blood pressure between the two groups. The survival rates for both groups were calculated using the Kaplan-Meier method. All tests were expressed as two-tailed, and a *P* value of <0.05 was considered statistically significant.

## Results

In the two hospital databases, we found 126,931 patients who underwent cardiac catheterization at the Department of Internal Medicine, University Hospital of Saarland, and HELIOS Hospital Wuppertal, Witten/Herdecke University Medical Center, Germany, during the study period from 2004 to 2013. A total of 192 (0.15%, mean age 91.45±1.75 years, 97 females [50.52%]; 95% confidence interval [CI], 0.0013–0.0017) patients over 90 years of age with a cardiac catheter met the inclusion criteria for this trial. A total of 175 (91.15%, mean age 91.51±1.80 years, 74 females [42.29%]; 95% confidence interval [CI], 0.87–0.95) patients over 90 years of age had CAD (study group); in 17 patients (8.85%, mean age 90.77±0.88 years, 10 females [58.82%]; 95% CI, 0.05–0.13), CAD was excluded by means of cardiac catheter (control group). We found a higher prevalence of CAD in males, but without increased risk (1.4450 odds ratio; 95% CI, 0.5261–3.9687; P = 0.4752).

FRSs did not differ between very elderly people over 90 years of age compared with and without CAD (P = 0.3792, Table 1). The calculation of Risk Scores 0–5 including one point for each cardiovascular risk factor showed statistical difference only for risk score 3 with statistical difference (P = 0.0244) compared between both study groups (Table 1). A statistical difference was also found in systolic blood pressure with higher levels in the group without CAD with statistical difference (P = 0.465). The levels of cholesterol, HDL, and BMI were statistically no different between the two groups (Table 1). The duration of hospital stays showed no statistical significance between the two groups (Table 1).

**Table 1:** Comparison of demographic data, duration of hospital stay, cholesterol, high sensitivity lipoproteins, systolic blood pressure, Framingham Risk Score, and Risk Score between elderly people over 90 years of age with and without CAD.

	CAD (%)	Without CAD (%)	P value
Number of patients (N = 192)	175 (91.15)	17 (8.85)	
Male	88 (50.29)	7 (41.18)	0.4733
Female	87 (49.71)	10 (58.82)	0.4733
BMI (kg/m^2^)	25.59±3.57	25.67±25.65	0.9205
Body height (cm)	166.31±8.85	163.13±8.36	0.1852
Body weight (kg)	70.38±10.61	68.27±11.58	0.4676
Duration of hospital stay (day)	6.02±8.03	6.29±6.21	0.8877
Framingham Score (%)	18.70±9.16	15±8.60	0.3792
Cholesterol (mg/dl)	168.89±42.21	196±27.40	0.1579
HDL (mg/dl)	52.96±20.86	59.6±13.48	0.4854
Systolic blood pressure (mmHg)	140.74±26.08	154.73±23.25	**0.0465**
Risk Score 0	16 (9.14)	3 (17.65)	0.2623
Risk Score 1	40 (22.86)	7 (41.18)	0.0935
Risk Score 2	60 (34.29)	3 (17.65)	0.163
Risk Score 3	41 (23.43)	0	**0.0244**
Risk Score 4	12 (6.86)	1 (5.88)	0.8786
Risk Score 5	6 (3.43)	0	0.4379

**Abbreviations:** CAD: coronary artery disease; BMI: body mass index; HDL: high sensitivity lipoproteins. **Notes:** Significant *P* values are shown in bold.

Following the results of this study, we identified a five-fold higher cardiovascular risk of developing CAD in patients over 90 years of age with arterial hypertension (P = 0.0035, Table 2). Very elderly diabetics had a three-fold, those with hypercholesterolemia had a one-fold, and very elderly former smokers had a two-fold increased risk of developing CAD, but without a statistically significant difference (Table 2). Neither group was distinguished statistically according to the number of subjects of normal weight, although most of the patients in the study population were not overweight (Table 2).

**Table 2:** Comparison of traditional risk factors for CAD in very elderly people over 90 years of age with and without CAD.

	Elderly>90 years of age				
Risk factors	CAD (n = 175) (%)	Without CAD (n = 17) (%)	Odds ratio	95% CI	P value
Hypertension	147 (84)	9 (52.94)	4.6667	1.6584–13.1318	**0.0035**
Diabetes	48 (27.43)	1 (5.88)	6.0472	0.7805–46.8540	0.0849
Hypercholesterolemia	17 (9.71)	1 (5.88)	1.7215	0.2148–13.7984	0.6090
Hyperlipidemia	47 (26.86)	3 (17.65)	1.7135	0.4712–6.2312	0.4136
Obesity	22 (12.57)	3 (17.65)	0.6710	0.1784–2.5236	0.5550
Smoker	6 (3.43)	0	1.3422	0.0725–24.8432	0.8433
Former smoker	14 (8)	0	3.1424	0.1796–54.9851	0.4330

**Abbreviations:** CAD: coronary artery disease; CI: confidence interval. **Notes:** Significant *P* values are shown in bold.

The largest group in the study group had 3-vessel CAD, followed by 2-vessel CAD, and the small group 1-vessel CAD (Table 3). A comparison of the traditional cardiovascular risk factors with the number of coronary arteries that were afflicted with CAD showed no statistical difference (Table 3).

**Table 3:** Comparison of traditional cardiovascular risk factors in different forms of coronary artery disease.

	Coronary artery disease			
Risk factors	1-vessel (n = 26)(%)	2-vessel (n = 53)(%)	3-vessel (n = 96)(%)	P value
Hypertension	19 (73.08)	45 (84.91)	83 (86.46)	0.2433
Diabetes	4 (15.38)	12 (22.64)	32 (33.33)	0.1320
Hypercholesterolemia	4 (15.38)	5 (9.43)	8 (8.33)	0.5620
Hyperlipidemia	3 (11.54)	14 (26.42)	30 (31.25)	0.1290
Obesity	4 (15.38)	3 (5.66)	15 (15.63)	0.1748
Smoker	1 (3.85)	1 (1.89)	4 (4.17)	0.8601
Former smoker	0	3 (5.66)	11 (11.46)	0.1450

Only for hypertension did we find a statistically significant difference after comparing the tested traditional cardiovascular risk factors with the clinical manifestation of CAD, such as stable angina or ACS (P<0.0001, Table 4). We also found no statistically significant difference between risk factors and the acute comorbidities in the two groups (Table 5). Cases with a negative outcome were found numerically and exhibited a statistical difference in acute comorbidities such as syncope, falls, and attacks of gout (Table 5). These acute comorbidities showed no increased risk for CAD.

**Table 4:** Comparison of cardiovascular risk factors with stable angina pectoris and acute coronary syndrome in very elderly people over 90 years of age with CAD.

	Coronary artery disease				
Risk factors	Stable angina (n = 30)(%)	Unstable angina (n = 121)(%)	NSTEMI (n = 61)(%)	STEMI (n = 47)(%)	P value
Hypertension	30 (100)	99 (81.82)	52 (85.25)	24 (51.06)	**<0.0001**
Diabetes	8 (26.67)	31 (25.62)	17 (27.87)	5 (10.64)	0.1429
Hypercholesterolemia	3(10)	18 (14.88)	9 (14.75)	4 (8.51)	0.6594
Hyperlipidemia	8 (26.67)	30 (24.79)	18 (29.51)	6 (12.77)	0.2186
Obesity	3 (10)	16 (13.22)	9 (14.75)	3 (6.38)	0.5459
Smoker	2 (6.67)	3 (2.48)	2 (3.28)	0	0.3618
Former smoker	3 (10)	10 (8.26)	6 (9.84)	2 (4.26)	0.722

**Abbreviations:** NSTEMI: non-ST segment elevation myocardial infarction; STEMI: ST segment elevation myocardial infarction. **Notes:** Significant *P* values are shown in bold.

**Table 5:** Comparison of acute illnesses in patients with and without CAD.

Cardiovascular diseases	CAD (n = 175) (%)	Without CAD (n = 17) (%)	Odds ratio	95% CI	P value
Acute heart failure	103 (58.86)	9 (52.94)	1.2716	0.4683–3.4526	0.6373
Anemia	8 (4.57)	0	1.7761	0.0983–32.0995	0.6973
Cardiac arrhythmia	62 (35.42)	8 (47.06)	0.6173	0.2267–1.6804	0.3451
Cardiac decompensation	18 (10.29)	3 (17.65)	0.5350	0.1402–2.0412	0.3599
Circulatory collapse	1 (0.57)	0	0.0118	0.0118–7.6681	0.4672
Derailed blood pressure	8 (4.57)	0	1.7761	0.00983–32.0995	0.6973
Shock	4 (2.29)	0	0.9184	0.0475–17.7742	0.9551
Syncope	1 (0.57)	4 (23.53)	0.0187	0.0019–0.1795	**0.0006**
**Pulmonary diseases**					
Acute respiratory failure	2 (1.14)	1 (5.88)	0.1850	0.0159–2.1532	0.1778
Aspiration pneumonia	1 (0.57)	0	0.3009	0.0118–7.6681	0.4672
Bronchopulmonary infection	3 (1.71)	1 (7.14)	0.2791	0.0274–2.8411	0.2841
Pneumonia	2 (1.14)	0	0.5043	0.0233–10.9290	0.6627
Pulmonary edema	4 (2.89)	0	0.9184	0.0475–17.4442	0.9551
**Gastrointestinal diseases**					
Duodenal ulcer	1 (0.57)	0	0.3009	0.0118–7.6681	0.4672
Gastritis	2 (1.14)	0	0.5043	0.0233–10.9290	0.6627
Gastrointestinal bleeding	2 (1.14)	0	0.5043	0.0233–10.9290	0.4672
Hyperglycemia	1 (0.57)	0	0.3009	0.0118–7.6681	0.4672
Reflux esophagitis 2 (1.14)	0	0.5043	0.0233–10.9290	0.4672	
**Kidney diseases**					
Acute kidney injury	3 (1.71)	0	0.7101	0.0352–14.3174	0.8233
Acute urinary tract infection	10 (5.71)	1 (5.88)	0.3009	0.0118–7.6681	0.4672
Macrohematuria	3 (1.71)	0	0.7101	0.0352–14.3174	0.8233
Water-electrolyte imbalance	2 (1.14)	0	0.5043	0.0233–10.9290	0.4672
**Thyroid diseases**					
Hyperthyroidism	1 (0.57)	0	0.3009	0.0118–7.6681	0.4672
Hypothyroidism	5 (2.86)	0	1.1290	0.0599–21.2845	0.9354
**Other conditions**					
Acute stroke	1 (0.57)	0	0.3009	0.0118–7.6681	0.4672
Abscess	1 (0.57)	0	0.3009	0.0118–7.6681	0.4672
Dizziness	2 (1.14)	0	0.5043	0.0233–10.9290	0.4672
Fall	0	1 (5.88)	0.0313	0.0012–0.8004	**0.0362**
Attack of gout	0	1 (5.88)	0.0313	0.0012–0.8004	**0.0362**
Delirium	2 (1.14)	1 (5.88)	0.1850	0.0159–2.1532	0.1778

**Abbreviations:** CAD: coronary artery disease; CI: confidence interval. **Notes:** Significant *P* values are shown in bold.

Chronic comorbidities exhibited no increased risk for CAD (Table 6). We found cases with negative outcomes with a statistically significant difference in terms of chronic lumbago and pacemakers. However, chronic lumbago and patients with pacemakers demonstrated no increased risk for CAD (Table 6).

**Table 6:** Comparison of chronic comorbidities in elderly people over 90 years of age with and without CAD.

Cardiovascular diseases	CAD (n = 175) (%)	Without CAD (n = 17) (%)	Odds ratio	95% CI	P value
Aneurysm	1 (0.57)	1 (5.88)	0.0920	0.0055–1.5408	0.0970
Cardiomyopathy	4 (2.29)	0	0.9184	0.0475–17.7742	0.9551
Carotid stenosis	3 (1.71)	1 (5.88)	0.2791	0.0274–2.8411	0.2810
Cor pulmonale	9 (5.14)	2 (11.76)	0.4066	0.0804–2.0563	0.2765
Hypertensive heart disease	29 (16.57)	4 (23.53)	0.6455	0.1965–2.1207	0.4708
Pacemaker	20 (11.43)	5 (29.41)	0.3097	0.0988–0.9707	**0.0443**
Peripheral arterial occlusive disease	17 (9.71)	0	3.8644	0.2226–67.0875	0.3533
State after syncope	3 (1.71)	0	0.7101	0.0352–14.3174	0.8233
Cardiac valvular defect	50 (28.57)	8 (47.06)	0.4500	0.1643–1.2322	0.1202
Chronic venous insufficiency	1 (0.57)	0	0.3009	0.0118–7.6681	0.4672
Varicose veins	2 (1.14)	0	0.5043	0.0233–10.9290	0.6627
State after bypass surgery	10 (5.71)	0	2.2205	0.1247–39.5395	0.5871
**Pulmonary diseases**					
Chronic obstructive pulmonary disease	12 (6.86)	2 (11.76)	0.5521	0.1129–2.7012	0.4639
Emphysema	2 (1.14)	0	0.5043	0.0233–10.9290	0.6627
Obstructive sleep apnea syndrome	1 (0.57)	0	0.3009	0.0118–7.6681	0.4672
State after tuberculosis	2 (1.14)	0	0.5043	0.0233–10.9290	0.6627
**Gastrointestinal diseases**					
Appendectomy	1 (0.57)	0	0.3009	0.0118–7.6681	0.4672
Cholecystectomy	16 (9.14)	1 (5.88)	1.6101	0.2002–12.9484	0.6543
Colonic diverticula	4 (2.29)	0	0.9184	0.0475–17.7742	0.9551
Gallbladder disease	1 (0.57)	0	0.3009	0.0118–7.6681	0.4672
Gastric carcinoma	6 (3.43)	0	1.3422	0.0725–24.8432	0.8433
Liver cysts	1 (0.57)	0	0.3009	0.0118–7.6681	0.4672
Pancreatic disease	1 (0.57)	0	0.3009	0.0118–7.6681	0.4672
Splenectomy	1 (0.57)	0	0.3009	0.0118–7.6681	0.4672
State after bowel surgery	3 (1.71)	0	0.7101	0.0352–14.3174	0.8233
State after hepatitis	3 (1.71)	0	0.7101	0.0352–14.3174	0.8233
State after hernia operation	4 (2.29)	0	0.9184	0.0475–17.7742	0.9551
State after gastric surgery	4 (2.29)	0	0.9184	0.0475–17.7742	0.9551
**Kidney diseases**					
Chronic renal failure	45 (25.71)	5 (29.41)	0.8308	0.2774–2.4883	0.7404
Contracted kidney	1 (0.57)	0	0.3009	0.0118–7.6681	0.4672
Diabetic nephropathy	3 (1.71)	0	0.7101	0.0352–14.3174	0.8233
Nephrectomy	3 (1.71)	0	0.7101	0.0352–14.3174	0.8233
Renal adenoma	2 (1.14)	0	0.5043	0.0233–10.9290	0.6627
Renal cysts	5 (2.86)	0	1.1290	0.0599–21.2845	0.9354
State after kidney stones	1 (0.57)	0	0.3009	0.0118–7.6681	0.4672
**Diseases of the genitourinary system**					
Benign prostate hyperplasia	4 (2.29)	1 (5.88)	0.3743	0.0394–3.5526	0.3920
Hysterectomy	4 (2.29)	0	0.9184	0.0475–17.7742	0.9551
Prostate cancer	3 (1.71)	1 (12.50)	0.2791	0.0274–2.8411	0.2810
Prostatectomy	3 (1.71)	0	0.7101	0.0352–14.3174	0.8233
State after bladder carcinoma	2 (1.14)	0	0.5043	0.0233–10.9290	0.6627
**Thyroid diseases**					
Struma	2 (1.14)	0	0.5043	0.0233–10.9290	0.6627
Strumectomy	2 (1.14)	1 (5.88)	0.1850	0.0159–2.1532	0.1778
**Nervous system disorders**					
Chronic lumbago	0	1 (5.88)	0.0313	0.0012–0.8004	**0.0362**
Disc herniation	2 (1.14)	0	0.5043	0.0233–10.9290	0.6627
Polyneuropathy	4 (2.29)	1 (5.88)	0.3743	0.0394–3.5526	0.3920
Parkinson disease	6 (3.43)	0	1.3422	0.0725–24.8432	0.8433
Restless legs syndrome	3 (1.71)	0	0.7101	0.0352–14.3174	0.8233
Spinal canal stenosis	2 (1.14)	1 (5.88)	0.1850	0.0159–2.1532	0.1778
Status after stroke	12 (6.86)	1 (5.88)	1.1779	0.1437–9.6544	0.8787
**Orthopedic disorders**					
Osteoarthritis	9 (5.14)	3 (17.65)	0.2530	0.0614–1.0425	0.0571
Osteoporosis	5 (2.86)	1 (5.88)	0.4706	0.0518–4.2786	0.5033
Rheumatism	2 (1.14)	0	0.5043	0.0233–10.9290	0.6627
**Psychiatric disorders**					
Alzheimer disease	1 (0.57)	0	0.3009	0.0118–7.6681	0.4672
Dementia	4 (2.29)	1 (5.88)	0.3743	0.0394–3.5526	0.3920
Depression	3 (1.71)	0	0.7101	0.0352–14.3174	0.8233
**Ear, nose, and throat diseases**					
Nasal polypectomy	1 (0.57)	0	0.3009	0.0118–7.6681	0.4672
Tonsillectomy	1 (0.57)	0	0.3009	0.0118–7.6681	0.4672
**Skin disorders**					
Allergy	1 (0.57)	0	0.3009	0.0118–7.6681	0.4672
Psoriasis	1 (0.57)	0	0.3009	0.0118–7.6681	0.4672
State post-herpes zoster	1 (0.57)	0	0.3009	0.0118–7.6681	0.4672
**Ophthalmologic diseases**	13 (7.43)	1 (5.88)	1.2840	0.1576–10.4623	0.8154
**Gynecological disorders**					
Status after breast cancer	3 (1.71)	0	0.7101	0.0352–14.3174	0.8233

**Abbreviations:** CAD: coronary artery disease; CI: confidence interval. **Notes:** Significant *P* values are shown in bold.

There were six (3.43%, 4 [66.67%] females; 95% CI, 0.0073–0.0613) deaths in the study group and no deaths in the control group (P = 0.4379). Thus, the survival rate was 96.57% (95% CI, 0.94–0.99) in the study group and 100% in the control group.

## Discussion

Past researchers have assumed that the incidence of acute myocardial infarction increases with advancing aging [34], [35]. According to the results of this study, after confirmation by cardiac catheterization, the FRS had an insufficient predictive value for CAD in very elderly people over 90 years of age with CAD. In this study, the assessment tool that was used to estimate 10-year risk after having a heart attack considered age, sex, total cholesterol, HDL, systolic blood pressure, smoking status, and whether patients were currently under medication for hypertension. However, age is in and of itself the strongest predictor of CAD.

For this reason, in one study, researchers examined the possibility of the prediction value of a risk factor on the prevalence of CAD to vary over a wide range of ages from middle age to old age [36]. The positive association between hypertension and CAD decreased considerably with age, primarily due to the significantly increased risk of CAD in elderly men without hypertension. The outcomes of total cholesterol on CAD also appeared to decrease with age, although variations were not statistically significant. In contrast, men with diabetes had a dependable two-fold additional risk of CAD transversely across all age groups, while a positive relationship with body mass index in younger men became negative in those who were the oldest. Due to the occasional smoking amongst the elderly, the relationship between smoking and CAD deteriorated with age. The results of this study suggest that changes in risk factor effects on the incidence of CAD with advancing age may require efficient approaches for CAD prevention as people age [35]. The influence of age on the incidence of CAD was not investigated in our study. Overall, the size of the study population in our study over the course of nearly 10 years was small. Therefore, we included the data of two hospitals in this study. This may be due to the patients' biological age, because with increasing age, the population decreases. We found that for patients over 90 years of age, only those with hypertension had a high risk for CAD. Traditional cardiovascular risk factors such as progressing age, diabetes mellitus, hypertension, dyslipidemia, smoking, and obesity are known to have a relationship with CAD [36], [37].

Veeranna et al. reported that age and male sex, but not hypertension or dyslipidemia, represented an increased risk for CAD. Only diabetes was an independent predictor of CAD, and smoking was associated with the occlusion of the left main trunk artery of the heart in their study [38]. This was quite different from our outcome, as we found a high risk of CAD in very elderly people with hypertension. Diabetes increased the risk of developing CAD, but without statistical significance, and smoking presented absolutely no increased risk for CAD in our analysis. While the number of male patients with CAD was slightly increased in our study, we could not find a statistically significant difference in sex regarding the risk for CAD.

In one study, researchers examined the influence of advancing age on clinical presentation and hospital reports in a large sample of patients with STEMI [39]. There was a significant converse relationship between age and the probability of presenting with STEMI. For each period of life, the probability of presenting with STEMI decreased. Noticeably fewer elderly patients were frequently treated by cardiologists, they were not examined as thoroughly, and when presenting with STEMI, a smaller number were likely to be treated with cardiac catheterization. Hospital mortality was augmented in the elderly. Fewer elderly patients presented with STEMI but had considerable in-hospital mortality rates; however, they had obviously fewer intensive treatments and investigations [39]. The amount of STEMI was decreased in our study as well. We did not investigate whether the very elderly were treated less often with cardiac catheterization and by cardiologists.

Although several scoring systems have been recommended to compute cardiovascular risk factors, some information has been lacking on significant variables such as family history of CAD or LDL cholesterol. Based on acute coronary events happening within 10 years of follow-up after enrollment into the Prospective Cardiovascular Münster (PROCAM) study [26], the authors of the study developed a Cox proportional hazards model using the following eight independent risk factors for CAD, graded in order of importance: age, LDL cholesterol, smoking, HDL cholesterol, systolic blood pressure, family history of previous myocardial infarction, diabetes mellitus, and triglycerides. They then developed a point scoring system created on the beta-coefficients of this model. The exactness of this point scoring system was similar to coronary event prediction when the continuous variables themselves were used. The scoring system accurately predicted detected coronary events [26]. Similar to this model, we could detect a statistical difference only for risk score 3 in the predictive value of traditional risk factors for CAD for very elderly people in our study when we calculated one point for each traditional risk factor, such as hypertension, diabetes, hypercholesterolemia, hyperlipidemia, obesity, and smoking. This risk assessment model was easy to calculate. Apparently, it makes no difference whether the exact numbers of serum lipids or their global viewpoint was used when calculating this risk factor. Because the risk factor of a family history of previous myocardial infarction cannot be influenced, we did not consider this established risk factor of family history for CAD when evaluating the very elderly in our study.

The question of the use of risk assessment for the primary prevention of CAD remains controversial. The validity of the FRS was assessed in a previous study [40]. Comparisons of prediction models and reality in tertiles were performed, and the individual survival functions were calculated. The mean risk for men was increased. Cardiovascular disease events happened in the highest risk tertiles. The negative predictive values in both sexes were noteworthy, and the specificity in women and sensitivity in men were high when their risk for cardiovascular disease was high. This model overestimated the risk in older women and in middle-aged men. The cumulative probability of individual survival by tertiles was significant in both sexes [40]. The results of this study warrant the reclassification of FRS.

Rodondi et al. also reached the conclusion that the FRS miscalculates the risk for CAD in the elderly, mainly in women [27]. They proposed that traditional risk factors best predict CAD. We detected no sex differences in the mean study population in very elderly people over 90 years of age with and without CAD in our study.

In one study, classic risk factors' validity was examined with several new biomarkers in predicting cardiovascular mortality in the very elderly from the general population with no history of CAD [40]. All classic risk factors were comprised in the FRS as well as serum concentrations of the biomarkers homocysteine, folic acid, C reactive protein, and interleukin 6 [40]. Classic risk factors did not forecast cardiovascular mortality when used in the FRS. Of the novel biomarkers investigated, homocysteine had the greatest predictive value. The inclusion of some additional risk factors or a combination of factors into the homocysteine prediction model did not increase its discriminative value. In very elderly people with no history of CAD, only serum levels of homocysteine were able to precisely detect those at high risk of cardiovascular mortality, whereas classic risk factors incorporated into the FRS did not [40]. Further investigations are warranted to confirm these findings.

Other findings have suggested that the FRS and PROCAM should not be carried out to calculate the absolute CAD risk of middle-aged men without any CAD history because of a clear overestimation [41]. In our study, some FRSs overestimated the probability of CAD occurrence, mainly in the very elderly after excluding CAD by cardiac catheterization. In contrast, a small number very elderly people with CAD were underestimated after the calculation of FRSs in our study.

In previous studies, researchers found that risk factors could have supplementary predictive value outside of what the FRS can predict [42]. However, most of the results of the examined studies had mistakes in their design, methods, and descriptions that limit their reliability and validity [42]. While hypertension was treated by medication in the very elderly, our study showed that the risk for CAD was high. While diabetes has been found in nearly over one-quarter of elderly patients with CAD, about one-tenth had hypercholesterolemia in our study, without statistical significance. Neither hyperlipidemia nor obesity was an increased risk factor for CAD in the very elderly patients in our study population. The same result was reported by Kim et al. in relation to the elderly [43]. These results raise questions about the value of weight loss and diet for the prevention of CAD in the elderly.

The effects of smoking on mortality in the elderly population have been studied previously [44]. When comparing the mortality rates for older smokers, ex-smokers, and non-smokers, lower mortality was observed for non-smokers and former smokers than for older smokers [44]. We found more very elderly former smokers and smokers with CAD than non-smokers in our study, but this was not statistically significant.

However, it continues to be difficult to correlate CAD and atherosclerosis. Even when this was evaluated angiographically, the connection has not been well established, and previous studies have reported different and varying outcomes concerning the link between CAD and atherosclerosis [45]–[47]. The severity of CAD in the elderly seemed to correlate poorly with the prevalence of established traditional cardiovascular risk factors in our study.

There are probably reasons for the different risk profiles for CAD in very elderly people. The assessment and identification of cardiovascular risk factors for CAD in the elderly may be challenging for further investigations.

### Study limitations

In this study, the FRS and traditional risk factors for CAD in very elderly people in two departments of internal medicine were examined, but not in very elderly people with CAD in other medical departments. The FRS was calculated after an acute myocardial infarction and preformation of cardiac catheterization to confirm CAD under the assumption that the identified risk factors would have existed 10 years ago. Another limitation was that we were unable to identify very elderly patients with ACS who had not undergone cardiac catheterization for any reason. Moreover, aging itself was considered a risk factor for CAD in previous studies. It is also possible that the risk profiles change over the time among all age groups. The influence of lifestyle and diet in the traditional risk factors were not considered in the very elderly in this study. Therefore, it is difficult to identify the risk profile for CAD in very elderly people. Various causes of the development of CAD have been discussed in the current scientific-medical literature. Most of the very elderly patients had CAD. Therefore, the group of very elderly patients without CAD was small. For this reason, we conducted a two-center study to exclude a statistical error in the limited sample size in the group of very elderly patients without CAD.

## Conclusions

We were not able to demonstrate that the FRS has sufficient predictive value in patients over 90 years of age with CAD. In addition, the scoring system with a point for each risk factor for CAD did not have sufficient predictive power for CAD in very elderly people. However, established risk factors such as hypertension, diabetes, hyperlipidemia, obesity, and smoking should be carefully considered in the therapeutic management and prevention of CAD in very elderly people, in addition to treatment for acute and chronic comorbidities.
